# The Social Norm to Work and the Well-Being of the Short- and Long-Term Unemployed

**DOI:** 10.1007/s11205-017-1723-0

**Published:** 2017-08-09

**Authors:** Karlijn L. A. Roex, Jesper J. Rözer

**Affiliations:** 1 Department of Sociology, Max Planck Institute for the Study of Societies, Paulstr. 3, 50676 Cologne, Germany; 2 Department of Sociology, Amsterdam University, Postbus 15508, 1001 NA Amsterdam, The Netherlands

**Keywords:** Health, Long-term unemployed, Social norms, Social norm to work, Stigma, Unemployment, Well-being

## Abstract

Why are the unemployed particularly unhappy in some societies? According to the social norm theory of unemployment, the well-being of the non-employed is lower in countries with a strong social norm to work because of the greater stigma attached to unemployment. In this study, a social norm to work has been defined as the extent to which people expect others to work: do people think the unemployed should take any job they are offered, or should they have a right to refuse? The combined world and European values study and the European social survey were used to test the theory. Multilevel analyses show that—net of one’s own norm and other measures of the social norm to work, such as one’s personal work ethic—the well-being of unemployed men is lower in countries with a strong social norm to work, in particular that of the long-term unemployed. Overall, it appears that the social norm to work still weighs more heavily upon men than women.

## Introduction

Why does the extent to which unemployment is detrimental to well-being differ so strongly across societies (e.g. Gallie and Russell 1998; Stavrova et al. 2011; Whelan and McGinnity 2000)? According to the social norm theory of unemployment, these differences can be attributed to a social norm that people should work, also called ‘the social norm to work’. The stronger this norm, the more the unemployed are disqualified and exposed to social and internal sanctions, and correspondingly the lower their well-being will be (Clark 2003; Stam et al. 2016; Stavrova et al. 2011). However, empirical evidence for this claim is mixed, even between studies that use identical indicators (Clark 2003; Oesch and Lipps 2013; Stam et al. 2016; Stavrova et al. 2011).

A number of reasons can be given for this mixed evidence. First, the weak evidence for the social norm theory of unemployment can be attributed to the difficulty of measuring the social norm to work. Measures that are frequently used to study this norm are unemployment rates (Clark 2003; Oesch and Lipps 2013) and the degree of electoral support for cuts in unemployment assistance expenditure (Stutzer and Lalive 2004). However, these are very indirect measures of a social norm to work that can also be a proxy for macroeconomic conditions, such as an economic crisis or a country’s overall prosperity level. Moreover, communities do not necessarily derive social norms from behaviours or phenomena that occur more frequently (Elster 2007). Therefore, recent research has replaced these measures with a measure based on individuals’ level of work ethic (Stam et al. 2016; Stavrova et al. 2011). To measure the effect of the social norm to work on the social level, these authors have used the average endorsement of the work ethic of individuals for countries and assessed their impact while controlling for people’s individual level of work ethic. Through this approach, these studies aimed to measure the strength of a national social norm, while ruling out the confounding effects of people’s personal norms. However, we argue that deducing a strong social norm to work from a strong presence of the work ethic can be problematic. As will be discussed later, a strong average work ethic, as described by Weber (1920) and as operationalized by these previous studies (Stam et al. 2016; Stavrova et al. 2011), indicates a high prevalence of individuals who endorse a strong personal norm to work (i.e. imposed on themselves). It does not necessarily indicate a strong *social* norm (i.e. imposed on others) (Elster 2007). Personal norms are only social norms if we impose them on others rather than only on ourselves (Elster 2007). A high average work ethic in a country does not necessarily imply that people expect others to behave in conformity with their personal norm. Thus, more direct and alternative measures are needed to see to what extent the social norm to work theory holds.

The second reason for this mixed evidence is that previous work has not taken into account the different implications of a social norm to work for the short- and long-term unemployed. The general theoretical conjecture is that a strong social norm to work in a society, internalized by individuals, will lead to internal or external sanctions on the unemployed, reducing their well-being. This would be the case regardless of the specific conditions and type of unemployment, which is surprising because there are several theoretical reasons to expect that the social norm to work would have different implications for short- and long-term unemployment. For instance, in societies with a strong norm to work, people may be relatively sympathetic toward the short-term unemployed, recognizing that it takes some time to find a new job. Another reason why the short-term unemployed are more socially accepted is that it is easier for those affected to conceal their unemployment as compared to the long-term unemployed. Hence, because previous cross-national work has grouped the short- and long-term unemployed together, it has likely underestimated the effects of a social norm to work (Gallie and Russell 1998; Stam et al. 2016; Stavrova et al. 2011).

Thirdly, the weak effects in favour of the social norm theory of unemployment can be accounted for by looking at the groups to which previous studies limited themselves. Most studies focus only on the unemployed (e.g. Helliwell and Huang 2014; Russell et al. 2013; Stavrova et al. 2011). However, a recent study (Stam et al. 2016) shows that other groups are affected as well. This can be explained by the presence of ‘hidden unemployment’ in societies with a strong social norm to work. In such societies, respondents that are in fact unemployed may prefer to avoid the disqualifying ‘unemployed’ label. Rather, they may self-classify as a homemaker, (early) retiree or disabled, even though others in their milieu think that they should be working (McFadyen 1995; Stam et al. 2016). Hence, while studying the social norm theory of unemployment, other non-employed groups than the unemployed should be taken into account.

The present study retests the social norm to work thesis while taking into account a more direct measure of the social norm to work, and it explores the differences between the short-term unemployed, the long-term unemployed, and other economically non-active groups. The present measure of the social norm records the extent to which members of a society believe that the unemployed should take any job offered to them or whether they should have the opportunity to refuse. Hence, it specifically examines whether individuals expect others to work whenever possible, and thus whether they impose this norm on others. In this way, we examine a crucial aspect of social norms, namely their social character. This measure correlates weakly with other measures of the social norm to work, such as the popular work ethic (*r* = .05, *p* = .659 on the national level and *r* = 17, *p* < .001 on the individual level) and hence provides a new test of the social norm theory of unemployment. An advantage of this measure is its availability over a longer time period and larger number of countries. This is crucial because a social norm to work is a national characteristic and hence a large number of countries (over time) are needed to test its effects. Furthermore, as we will discuss later in greater detail, it correlates as expected with other measures, and it affects the well-being of the unemployed, even after controlling for the alternative measures.

Our data on the norm to work originate from the *European Values Study* (EVS) and *World Values Survey* (WVS) dataset. With the WVS data, this study examines the effect of the norm to work between 1990 and 2009. As a second dataset this study employs the *European Social Survey*. Although this second dataset includes fewer countries and cannot control for whether individuals themselves think that others should work, it allows us to make a distinction between the long- and short-term unemployed.

Following recent studies, we aim to disentangle the effect of the social norm to work on both the social and the individual level by including both a variable measuring individuals’ endorsement of the norm as well as an aggregated version of this variable (Stam et al. 2016; Stavrova et al. 2011). Furthermore, as is done in previous work, gender interactions will be taken into account because men appear to be more strongly affected by a social norm to work than women (Oesch and Lipps 2013; Stam et al. 2016; Van der Meer 2014).

## The Social Norm to Work Theory of Unemployment

Unemployment imposes both financial and non-pecuniary costs on individuals (Latif 2010; Van der Meer 2014; Winkelmann and Winkelmann 1998). An important consequence of unemployment is a reduction in financial resources (Hauser and Nolan 2000). Financial resources are needed to buy all kinds of necessities, such as accommodation, heating or food. However, even when these necessities are guaranteed, the unemployed can still face relative deprivation when they cannot obtain the conditions of life that allow them to play the roles expected of them by society (Townsend 1993: 36). Hence, not being able to go out, buy a car or send one’s children to a sports club can seriously limit one’s well-being when these are ‘common’ goods (Adjaye-Gbewonyo and Kawachi 2012; Townsend 1993).

However, the benefits of being employed extend far beyond the earning of income. For example, it makes people feel that they are contributing something to society, increases their self-confidence, enlarges their social networks, increases their social and on-the-job skills, boosts their social status, and results in social approval (Kampen et al. 2013; Theodossiou 1998; Van der Meer 2014). These non-pecuniary aspects of employment explain why the detrimental effects of unemployment usually hold even when income is controlled for (Latif 2010; Van Der Meer 2014; Winkelmann and Winkelmann 1998).

According to the social norm theory of unemployment, a social norm to work increases the non-pecuniary costs of being unemployed (Clark 2003; Stam et al. 2016; Stavrova et al. 2011). In societies with a strong social norm to work, the unemployed face a greater risk of social sanctions and stigmatization. They are discredited and disqualified from ‘full social acceptance’ (Goffman 1963) and are likely to be excluded from social activities or be gossiped about. This may leave unemployed people feeling inferior and ashamed (Goffman 1963; Stam et al. 2016; Stavrova et al. 2011). As Goffman noted (1990[1963]: 28): ‘*How hard and humiliating it is to bear the name of an unemployed man’*.

Although the enforcement of social norms may have been faded in modern Western societies (Elias 1982; Heckhausen 1999), external social sanctions against the unemployed continue to be widespread today (e.g. Gustafson 2009; Stam et al. 2016) and may have even intensified with neoliberalism. Social norms can be regarded as social facts (Durkheim 1895) that exist in the community and create a social context in which certain behavioural expectations are imposed on individuals, independent of whether people personally agree with the norm. It is the context of the expectation that matters to people, because aversive social sanctions are attached to non-compliance. Hence, even those unemployed people who do not feel personally attached to the norm to work may feel the non-pecuniary cost of unemployment when living in a society that disapproves of them for not working.

Besides being subject to social sanctions, people may also internalize social norms. Norm non-compliance then activates an internal self-sanction system that metes out punishment, leading, for example, to feelings of guilt that may lower well-being (Coleman 1990; McAdams 1997).

The negative feelings of guilt or shame induced by the internal and external sanctions reduce overall well-being. Structural negative feelings can create chronic stress. By definition, chronic stress is a feeling of emotional discomfort. It also consumes energy, which gives the body less time to recover and comes at the expense of other important functions such as muscle capacities, brain function, and defence mechanisms (Carter and Barrett 2006; Hulme and Shepherd 2003). Chronic stress can result in serious health conditions, including anxiety, headaches, insomnia, muscle pain, high blood pressure, and a weakened immune system. It can also contribute to the development of major illnesses, such as heart disease and depression (Baum and Polsusnzy 1999; Thoits 2010). These factors together seriously hamper people’s well-being. Furthermore, by consuming energy, chronic stress may limit the unemployed in improving their overall life and financial situation, and in this way, chronic stress may indirectly result in an additional reduction of well-being (Carter and Barrett 2006; Hulme and Shepherd 2003). Moreover, a social norm to work can also raise the financial costs of being unemployed by reducing the willingness of family and friends to offer financial support. This may lower the capability of the unemployed to obtain financial resources—for instance through their social network—even more.

### The Short- and Long-Term Unemployed

Almost everybody experiences unemployment at one point in their life, irrespective of their education, socioeconomic background, or gender. In particular in modern societies, in which temporary contracts are becoming increasingly common, people are often unemployed for some time (Eichorst and Marx 2011; De Lange et al. 2012; Kim 2013). People can therefore hardly be blamed for it. However, in virtually all societies, the unemployed are expected to be actively looking for a job because they often receive social benefits. The longer people remain unemployed, the more they are suspected of deliberately choosing to be unemployed (Murray 2012; Oesch and Lipps 2013; Theodore 2007). Such conceptions may lead to strong social disapproval on the part of the public, especially towards the long-term unemployed. Therefore, it is expected that a strong social norm to work in society has stronger harmful effects on the well-being of the long-term unemployed than on that of the recently unemployed.

Even those long-term unemployed who ‘choose’ to be unemployed inevitably face the non-pecuniary cost of social disapproval. The higher social disapproval suffered by the long-term unemployed is indicated by their higher degree of social isolation, especially in north-western Europe (Paugam and Russell 2000). Moreover, in a Dutch study long-term unemployed respondents experienced disapproval and accusations of being work-shy (Kampen et al. 2013). Furthermore, the social networks of the long-term unemployed tend to consist predominantly of other long-term unemployed, barring them from effective sources of financial support or relevant job network relationships (Gallie 1999; Nordenmark 1999). This high segregation in their networks probably indicates social rejection on the part of working people, an oft-cited form of social sanction (Elster 2007; Stavrova et al. 2011).

### The ‘Hidden Unemployed’, Including the Economically Inactive

We have so far differentiated between the short- and long-term unemployed based on the length of their unemployment spells, but there is also a relevant differentiation between the unemployed and the economically inactive. Stam et al. (2016) showed that economically inactive disabled males in particular suffer a relatively large loss in well-being in societies with a strong norm to work. In contrast, a stronger norm to work did not lower the well-being of the unemployed in their study. The authors concluded that the norm to work particularly applies to the non-working disabled. This is surprising, since other studies have suggested that public resentment is strongest against the unemployed, more so than against economically inactive people such as the disabled (Furaker and Blomsterberg 2003; Van Oorschot 2006). Looking at the chronologic order in which different welfare arrangements have been introduced in Western countries, Van Oorschot (2006) argued that the sick, old and disabled are considered most ‘deserving’ and the unemployed most undeserving.

However, this may have changed with the increasing dominance of neoliberal thought in modern societies. These ideological shifts have led to reforms in capacity to work laws in which the inability of the disabled to work has been increasingly re-evaluated. More disabled people are driven into the group of the ‘undeserving’ unemployed while still characterizing themselves as disabled. The lack of consensus on the issue of whether the disabled are not deserving, has indeed contributed to a higher pressure on this group to work (Jeene et al. 2013).

An alternative interpretation of the previously puzzling findings with regard to disabled men points to the role of ‘hidden unemployment’. The ‘hidden unemployed’ are those who do not appear as unemployed in the data, but rather as economically inactive, even though they can work or are expected to do so by others. For example, many discouraged older unemployed may self-identify as having retired early (Buffel et al. 2017).

This tendency to self-identify with other employment statuses may be stronger when the social norm to work is stronger. Indeed, by self-classifying as something other than unemployed, one can largely avoid the strong stigma of unemployment (McFadyen 1995; Stam et al. 2016). Within societies with a strong norm to work, in particular individuals who are sensitive to this stigmatization may prefer to self-categorize as ‘homemaker’ or ‘in early retirement’, roles which are more socially accepted. However, for various reasons, it may be the case that these ‘hidden unemployed’ experience at least the same amount of negative well-being as a consequence of the strong social norm to work as the ‘non-hidden unemployed’. Firstly, fully concealing one’s unemployment may be difficult, and disapproval and shame are therefore hard to escape. Secondly, the hidden unemployed may suffer from guilt and self-stigmatization because they have internalized the social norm to work (McAdams 1997). Hence, it should be taken into account that a social norm to work not only affects the unemployed, but also can affect some of the unemployed people who classify as economically inactive. These people may lower the well-being of these further heterogeneous groups, among whom many may not identify as unemployed and who are not seen as unemployed by others.

## Data and Measurement

The individual-level data used in this study came from two datasets. The first dataset contains both the combined *World* (WVS*)* and *European Values Study* (EVS) (WVS 2016). It includes a number of good proxies for the social norm to work and is unique in the large number of countries covered. Moreover, it covers that large number of countries over a long period of time, which increases the power of the study. The second dataset we used is the *European Social Survey* (ESS), covering only European countries over the period from 2002 to 2008 (ESS 2015). Although this dataset does not include individual proxies for the social norm to work, it contains a more refined measure of employment status. This makes a better examination of the impact on the economically inactive possible, and it allows us to make the distinction between the long- and short-term unemployed.

### WVS and EVS

The WVS and the EVS share a common questionnaire and procedures for sampling and data collection. The data were collected through phone or face-to-face interviews on a sample generated through a multi-stage or stratified random sampling procedure. After we omitted country-years from the analysis because of a lack of data on a key variable, we had 134 country-year points^1^ (48 countries for the period from 1990 to 2009) at our disposal. The analysis focused on people of working age (21–65 years old, N = 142,833), because the norm to work applies mainly to people in this age group.

The individual measurements of this study are as follows. To describe their *subjective well*-*being*, individuals were asked to describe their well-being both through items that measured both their affective and cognitive reflections on their well-being (Veenhoven 2008). The ‘affective’ item asked individuals how happy they felt, and the ‘cognitive’ item asked them to compare their current life with their desired life. This latter item captures life satisfaction and was measured with the question ‘All things considered, how satisfied are you with your life as a whole nowadays?’ Answers ranged from dissatisfied (1) to satisfied (10). The ‘affective item’ captures happiness, and was measured with the question: ‘Taking all things together, how happy would you say you are?’, with answer categories ranging from very happy (1) to not happy at all (4). For the analyses the mean of both measures was calculated after happiness was rescaled to range from 0 to 9. The suitability of the measure for cross-national purposes can be questioned because it can be influenced by cultural conditions, which differ among countries. However, the main interest here is in the well-being *gap* between the unemployed and the employed within countries, and how this well-being gap differs between countries. Therefore, the results of this study are unlikely to be affected by cross-national differences in people’s understanding of the item (descriptive statistics are shown in Table 1).

**Table 1:** Descriptive statistics on individual and country characteristics, WVS/EVS data

	Min	Max	Male		Female	
			Mean	SD	Mean	SD
*Individual level*						
Subjective well-being	0	9	5.94	1.90	5.95	1.97
*Employment status*						
Full-time employed	0	1	.62		.43	
Unemployed	0	1	.09		.08	
Retired	0	1	.11		.11	
Homemaker	0	1	.00		.18	
Student	0	1	.03		.04	
Part-time employed	0	1	.04		.10	
Self-employed	0	1	.09		.04	
Other	0	1	.02		.02	
Age	21	65	41.67	12.71	42.02	12.61
*Education*						
0 Incomplete primary	0	1	.02		.03	
1 Primary	0	1	.11		.12	
2 Incomplete secondary	0	1	.13		.12	
3 Secondary vocational	0	1	.19		.15	
4 Incomplete sec univ	0	1	.12		.11	
5 Secondary univ	0	1	.20		.22	
6 Incomplete university	0	1	.11		.12	
7 University	0	1	.13		.13	
*Marital status*						
Married/living together	0	1	.67		.66	
Single	0	1	.25		.17	
Divorced/separated	0	1	.07		.10	
Widowed	0	1	.02		.07	
Income	0	1	5.83	2.79	4.72	2.79
Church attendance	0	7	2.42	2.35	2.99	2.38
Norm to work	1	10	6.15	2.89	6.08	2.89
Work ethic	1	5	3.59	.77	3.53	.78
*National level variables*						
GDP	2965.10	82,904.15	2128.36	12,197.66		
Unemployment rate	.70	33.80	8.45	4.82		
Norm to work	4.24	7.93	6.21	.75		
Replacement rate	2.02	72.31	37.12	14.01		
Work ethic	2.82	4.25	3.58	.28		

Number of females = 77,016 and males = 66,679

*Employment status* was measured as follows: respondents could indicate that they were a full-time employee, part-time employee, self-employed, unemployed, homemaker, student or retired, or specify their employment status as ‘other’.

The *social norm to work* of a society was calculated by aggregating the answers of all respondents within a given country-year on one item. The instructions were: ‘How would you place your views on this scale? 1 means you agree completely with the statement on the left, 10 means you agree completely with the statement on the right, or you can choose any number in between’, and the item was ‘People who are unemployed should have to take any job available or lose their unemployment benefits’ (1) to ‘People who are unemployed should have the right to refuse a job they do not want’ (10). The item was reverse-coded such that a high score (up to 10) represented a strong norm to work (‘they should take any job’) and a low score (toward 1) indicated a weak norm to work (‘right to refuse a job’). This measure thus captures the extent to which people in a society are expected by others to work. A higher share of people that impose this expectation on others will increase the vulnerability of the unemployed to external social sanctions. The norm to work is particularly weak in eastern European countries such as Montenegro (4.8) and Ukraine (5.1), and strong in the US (6.2), northern European countries such as Germany (6.5), and in Slovenia (7.3) and Italy (7.3).

We argue that this item measures a social norm to work because it reflects the tendency of individuals to opine that everybody should work if given the chance (‘should take any job’); it is an imperative that people impose on the generalized other (unemployed). This is what makes social norms *social*: they are behavioural expectations imposed on others (Elster 2007; Bicchieri 2006). Individuals who allow the unemployed to refuse any unwanted job they, do not impose a norm to work on others, while others may locate themselves somewhere between the two extreme answer options and adhere to a medium weak or strong social norm to work.

Additionally, the present study also looks at the individual endorsement of the social norm to work. This way, the influence of possible self-sanctioning among unemployed people can be taken into account. As a results, the effect of the social norm to work is independent of individual level preferences.

Across societies, the national measure correlates remarkably weakly with countries’ work ethic (which measure will be discussed later) (*r* = .05, *p* = .659) (using the same standard measure as Stam et al. 2016; Stavrova et al. 2011). This indicates that this measure taps into a distinct characteristic of the social norm to work and hence provided a completely new test of the social norm theory of unemployment in the present study. There are a number of reasons why the measure from the current study might tap a different dimension of the social norm to work.

First, the face validity of the current measure can be argued to be higher than that of work ethic due to its stronger incorporation of the aspect of social expectations. It questions whether others, i.e. the unemployed, should take any job.

The second reason this measure may be more appropriate is its availability over a longer time period and a larger number of countries than previous studies (e.g. Stavrova et al. 2011, who studied the period 1999–2009 and covered 55 country-years). This is crucial because a social norm to work is a national characteristic, and hence a large number of countries (over time) is needed to test its effects.

Thirdly, the measure correlates as expected with other individual and national characteristics. We find that it correlates with a right-wing political orientation (as can be expected since right-wing parties are stricter on the unemployment benefits (*r* = .08, *p* < .001; *r* = .18, *p* < .001 in West European countries in which right wing is more connected to liberal labour market policies); and that it correlates with the belief that immigrants are a strain on the welfare state (*r* = .12, *p* < .001). Furthermore, there are strong correlations with countries’ GDP (*r* = .40, *p* < .001), social expenditures (*r* = .35, *p* = .075) and unemployment level (*r* = −.26, *p* < .01). Whereas countries’ work ethic also correlates highly with social expenditures (*r* = .64, *p* = .115), it correlates with a lower GDP (*r* = −.41, *p* < .001) and a higher unemployment level (*r* = .30, *p* < .05). We find it more plausible that a social norm to work is stronger in wealthier countries and countries with low unemployment levels because individual attributions of unemployment may become more dominant during times of low unemployment (Buffel et al. 2017), although one could also argue that the social norm to work would be stronger in times of higher unemployment because of a higher strain on the welfare state. Moreover, the current measure correlates most strongly with the items underlying people’s work ethic that most directly relate to the unemployed, i.e. ‘people who do not work turn lazy’ (*r* = .14, *p* < .001 on the individual level and *r* = .01, *p* = .982 on the macro-level) and ‘work is a duty towards society’ (*r* = .15, *p* < .001 on the individual level and *r* = .41, *p* < .001 on the macro-level), while it correlates most weakly with the item that least relates to the unemployed, i.e. ‘to develop talents you need to have a job’ (*r* = .06, *p* < .001 on the individual level and *r* = −.05, *p* = .659 on the macro-level), as well as with the entire work ethic scale (r = .17, *p* < .001 on the individual level and *r* = .05, *p* = .699 on the macro-level).^2^

A clear weakness of the current measure is that it is only based on one item, as compared to five items representing countries’ level of work ethic. This also makes it difficult to assess its cross-cultural equivalence.

We used a number of individual and national-level *control variables* that are commonly known to be related to unemployment and well-being (Russell et al. 2013; Stavrova et al. 2011): age and age squared; education (ranging from 0, meaning uncompleted primary education, to 7, meaning university with degree); self-rated income level (running from 0, the lowest category, to 10, the highest category); marital status (married/living together, single, divorced/separated, widowed); and religiosity (‘how often do you attend religious services?’—with answer categories ranging from (0) ‘never’ to (7) ‘more than once a week’). Moreover, a control variable was added for people’s personal endorsement of the social norm to work. At the national level, the analyses controlled for GDP per capita and unemployment levels derived from World Bank data. Like some previous studies (e.g. Sjöberg 2010), the analyses controlled for a state’s welfare generosity by including the income replacement rate of unemployment insurance benefits, which was derived from OECD data.

### The European Social Survey

The ESS is a repeated cross-sectional survey in which individuals 15 years of age and older are interviewed face-to-face. In general, the ESS is considered to be of high quality, which is reflected in its relatively high response rates and many reliable and valid measurements. Additionally, samples are representative of the countries from which they are drawn (ESS 2016). Ultimately, the dataset we used contained information on 125,233 respondents living in 31 countries for 2002, 2004, 2006 and 2008. In sum, we had 74 country-year points at our disposal

We attempted to align the data as much as possible with the WVS/EVS, except for the more detailed measure of employment status (see Table 2 for descriptive statistics). For *subjective well*-*being*, again the mean score of people’s happiness and life satisfaction was used. For the item ‘Taking all things together, how happy would you say you are?’, respondents ranked their happiness on a scale from 0 (extremely unhappy) to 10 (extremely happy). Another item asked respondents: ‘All things considered, how satisfied are you with your life as a whole nowadays?’, with answer options ranging from 0 (extremely dissatisfied) to 10 (extremely satisfied). In this way, our analyses using the ESS dataset also provide a robustness check for the sensitivity of the findings since we converted a 4-points measure (happiness in the WVS/EVS) to a 10-point measure.

**Table 2:** Descriptive statistics on individual and country characteristics, ESS data

	Min	Max	Male		Female	
			Mean	SD	Mean	SD
*Individual level*						
Subjective well-being	0	10	6.95	1.96	6.95	2.04
Employment status						
Employed	0	1	.66		.46	
Retired	0	1	.09		.09	
Homemaker	0	1	.100		.32	
Student	0	1	.05		.05	
Non-working disabled			.03		.02	
Short-term unemployed	0	1	.03		.02	
Long-term unemployed	0	1	.02		.02	
Other			.02		.02	
Age	21	65	43.00	1.26	43.37	12.47
*Education*						
1 Less than lower sec	0	1	.05		.06	
2 Lower secondary	0	1	.13		.15	
3 Lower tier upper sec	0	1	.29		.23	
4 Upper tier upper sec	0	1	.22		.23	
5 Advanced voc. sub-degree	0	1	.09		.09	
6 Lower tertiary	0	1	.10		.13	
7 Higher tertiary	0	1	.12		.11	
*Marital status*						
Married/living together	0	1	.59		.60	
Separated	0	1	.02		.02	
Divorced	0	1	.07		.10	
Widowed	0	1	.02		.06	
Never married	0	1	.30		.22	
Income	−1.97	.214	.974	.95	.06	.98
Church attendance	1	7	2.321	1.41	2.70	1.51
*National level variables*						
GDP	5577.49	67,115.61	27,821.76	11,008.06		
Unemployment rate	2.60	19.90	7.11	3.28		
Norm to work	4.78	7.55	6.35	.60		
Replacement rate	19.40	71.89	4.89	15.72		
Work ethic	2.88	4.09	3.53	.26		

Number of females = 67,183 and males = 58,854

For employment status, people were asked: ‘Which of these descriptions applies to what you have been doing for the last 7 days?’. The following categories were distinguished: ‘employed (or self-employed)’, ‘unemployed for less than 2 years’, ‘unemployed more than 2 years’, ‘in education’, ‘permanently sick or disabled’, ‘retired’, ‘doing housework’, and ‘other’.

The *social norm to work* values derived from the EVS/WVS data were added to the ESS data as a contextual variable and assigned to the corresponding country-years. We entered the same control variables in the analyses of the ESS as in the EVS/WVS analyses. *Educational attainment* was measured on the ISCED scale, ranging from 1 (‘less than lower secondary’) to 7 (‘higher tertiary education’). Furthermore, we controlled for self-ranked *income* level (standardized score ranging from −1.49 to 1.73), *marital status* (married, separated, divorced, widowed, and never married), and *church attendance* (ranging from (1) ‘never’ to (7) ‘every day’). Unfortunately, it was not possible to control for people’s personal endorsement of the norm because the item used for the social norm to work in the EVS/WVS was not available in the ESS. On the national level, we again controlled for GDP, unemployment rate and replacement rate.

*Work ethic* was measured using the same measurement as in Stam et al. (2016) and Stavrova et al. (2011), using people’s endorsement of the following five statements: ‘Work is a duty towards society’, ‘People who don’t work turn lazy’, ‘It is humiliating to receive money without having to work for it’, ‘Work should always come first, even if it means less spare time’, and ‘To fully develop your talents, you need to have a job’.

## Methods

We conducted multilevel analyses because individuals were nested within countries and time periods (Snijders and Bosker 1999). This analysis strategy avoids the underestimation of standard errors due to the independence of observations. The models took into account the nesting of all potential levels, thereby avoiding any mismatch between the random and fixed parts of the models (Schmidt-Catran and Fairbrother 2016). Therefore, individuals were nested within country-years. Wave fixed effects were included to control for the potential influence of unspecified overall time trends that are related to differences in well-being as well as to the norm to work. Moreover, country fixed effects were if possible^3^ included to control for the potential influence of unspecified time-invariant country-characteristics that are related to well-being as well as to the norm to work. Hence, we examine variations (e.g. of the social norm to work) within countries over time and control for average changes across time.

To obtain meaningful effect estimates, the social norm to work was centred on the grand mean. In the cross-level interactions, the individual level variables were allowed to vary over country-years. Letting other variables vary over country-years did not alter the results. Missing data on the individual-level variables were imputed using multiple imputation. Linear interpolation was used to replace missing values on the national-level variables.

The analyses were built up as follows. Model 1 included the control variables and the effects of employment status, as well as the interaction effect between individuals’ own endorsement of the social norm to work and their employment status. Subsequenlty, Model 2 retested the social norm to work hypothesis (e.g. Stavrova et al. 2011; Stam et al. 2016), by using our new measure of the social norm to work. This was done by including the interaction effect between employment status and the macro-level norm to work. Model 3 added the income replacement rates of unemployment benefits to the model as an additional control variable. Although this measure may be better suited to holding the potential influence of welfare states constant, it also greatly reduced the number of available country-year points (toward 41 for the WVS/EVS and 80 for the ESS). Model 4, added countries’ ‘work ethic’ to the model (for 40 country-year points in the WVS and 74 in the ESS) to show to what extend the current measure has an independent effect on the unemployed above and beyond the effect of the previous measure of the social norm to work. Note that Model 3 and 4 are not directly comparable with Model 1 and 2, because of differences in sample size. Separate analyses were conducted for men and women to check the expectation of a stronger effect of unemployment for men.

## Results

### Results from the WVS and EVS Dataset

Table 3 presents the outcomes of the multilevel models based on the WVS/EVS dataset. Model 1 presents the main effects of the individual- and national-level variables. It shows that being unemployed has its well-known negative effect on well-being. For men, the effect is larger than for women (−.807 versus −.582 points, on a 10-point scale, both at *p* < .001). Interestingly, among men, those with ‘other’ employment status clearly have the lowest levels of well-being. The effects of unemployment or economic inactivity are not higher or lower among individuals who more strongly endorse the social norm to work. Moreover, personally subscribing to the norm to work does not affect individuals’ level of well-being, and neither does such personal norms influence the effect of individuals’ employment status on their well-being. Furthermore, both for men and women, the usual control variables have their expected effects on well-being: age has a u-shaped effect, and those who are married/living together have the highest levels of well-being. Church attendance, a higher income, and a higher education level are associated with a higher level of well-being. Countries’ higher wealth has a positive impact on general well-being, while this well-being is not affected by the strength of the norm to work in a country. The unemployment rate has a non-robust positive effect on well-being among men.

**Table 3:** Multilevel regression on well-being, WVS/EVS data

	Men				Women			
	M1	M2	M3	M4	M1	M2	M3	M4
Constant	−.492 (.003)***	−.492 (.003)***	−.477 (.005)***	.479 (.005)***	.519 (.003)**	.519 (.003)***	.476 (.005)***	.480 (.005)***
*Individual level* ^a^								
Employment status (ref = full-time)								
Unemployed	−.807 (.061)***	.322 (.335)	.755 (.605)	.933 (.813)	−.582 (.061)***	−.577 (.061)***	−.493 (.123)***	−1.584 (.667)*
Retired	−.292 (.085)**	−.856 (.316)**	.365 (.535)	.878 (.727)	−.123 (.066)^+^	−.119 (.066)^+^	−.159 (.130)	1.111 (.641)^+^
Housewife	−.137 (.328)	−.879 (1.119)	−4.641 (2.428)^+^	−1.477 (4.254)	.099 (.052)^+^	.104 (.052)*	.070 (.096)	−.487 (.519)
Students	.045 (.094)	.338 (.435)	2.348 (.824)**	.769 (1.134)	.049 (.092)	.065 (.091)	−.161 (.168)	−2.160 (.863)*
Part time	−.184 (.100)^+^	.408 (.392)	.187 (.874)	−.032 (1.211)	.118 (.066)^+^	.121 (.066)^+^	−.020 (.116)	−.217 (.582)
Self-employed	.083 (.068)	.629 (.335)^+^	1.043 (.538)^+^	1.111 (.699)	.067 (.090)	.067 (.090)	−.039 (.160)	−.405 (.775)
Other	−.894 (.119)***	−.412 (.528)	.354 (.987)	1.760 (1.338)	−.388 (.111)**	−.386 (.111)**	−.952 (.186)***	−2.426 (.872)**
Norm to work	.032 (.003)**	.032 (.003)***	.033 (.006)***	.031 (.006)***	.030 (.004)**	.030 (.004)***	.026 (.006)***	.023 (.007)**
Work ethic				.099 (.017)***				.051 (.016)**
*Individual level interactions*								
Norm to work*								
Unemployed	.005 (.008)	.007 (.008)	.009 (.017)	−.301 (.091)**	−.002 (.008)	−.001 (.008)	−.007 (.018)	−.006 (.018)
Retired	.010 (.011)	.007 (.011)	−.013 (.017)	−.079 (.078)	.002 (.008)	.001 (.008)	.006 (.017)	.007 (.016)
Housewife	−.016 (.049)	−.019 (.050)	.056 (.062)	.568 (.414)	−.003 (.006)	−.003 (.006)	−.005 (.012)	−.006 (.012)
Students	−.007 (.014)	−.007 (.014)	−.012 (.026)	−.373 (.125)**	.002 (.013)	.007 (.013)	.041 (.024)^+^	.045 (.025)^+^
Part time	.005 (.015)	.007 (.015)	.012 (.030)	.096 (.140)	−.015 (.009)^+^	−.014 (.009)	.003 (.015)	.006 (.015)
Self-employed	−.005 (.008)	−.004 (.008)	−.018 (.016)	−.115 (.077)	.003 (.012)	.001 (.012)	.013 (.022)	.019 (.023)
Other	.031 (.017)^+^	.033 (.017)^+^	.072 (.028)*	.231 (.162)	.024 (.016)	.026 (.016)	.058 (.025)*	.058 (.024)*
*National level variables* ^b^								
Norm to work	−.003 (.048)	.028 (.052)	−.476 (.328)	−1.285 (.524)*	.056 (.040)	.081 (.046)^+^	−.293 (360)	−2.381 (.522)***
Unemployment rate	.015 (.007)*	.015 (.007)*	.041 (.025)	−.030 (.046)	.009 (.006)	.009 (.006)	.035 (.028)	−.023 (.045)
Replacement rate			−.008 (.006)	−.004 (.452)			−.002 (.007)	−.004 (.005)
Work ethic				2.047 (.973)*				
*Cross-level interactions*								
Norm to work*								
Unemployed		−.185 (.054)**	−.262 (.094)**	−.301 (.091)**		−.084 (.051)^+^	−.214 (.120)*	−.313 (.091)**
Retired		.093 (.051)^+^	−.071 (.084)	−.079 (.078)		.061 (.045)	.091 (.111)	.099 (.075)
Housewife		.124 (.181)	.677 (.379)^+^	.568 (.414)		−.062 (.046)	.060 (.108)	.056 (.069)
Students		−.048 (.069)	−.349 (.126)**	−.373 (.125)**		−.160 (.064)**	−.169 (.140)	−.211 (.114)^+^
Part time		−.098 (.064)	−.078 (.138)	−.096 (.140)		−.060 (.049)	−.040 (.117)	−.012 (.083)
Self-employed		−.089 (.054)^+^	−.136 (.084)	−.115 (.077)		.061 (.061)	.208 (.133)	.149 (.106)
Other		−.079 (.085)	−.243 (.153)	−.231 (.162)		−.061 (.070)	.289 (.165)^+^	.287 (.152)^+^
Work ethic*								
Unemployed				.018 (.156)				.308 (.184)^+^
Retired				−.118 (.160)				−.354 (.175)*
Housewife				−.720 (.854)				.165 (.142)
Students				.479 (.223)*				.555 (.236)*
Part time				.093 (.240)				.048 (.164)
Self-employed				−.054 (.142)				.098 (.211)
Other				−.418 (.304)				.408 (.251)
Respondents	66,287	66,287	19,464	18,207	76,546	76,546	23,259	21,852
Country-years	134	134	41	38	134	134	41	38
Countries	48	48	30	30	48	48	30	30

Data are nested in country-years nested in countries. In Model 2 and 3 a random slope for employment status is included. Year fixed effects are included, but not presented

Unstandardized results, standard errors in parentheses

+ *p* < .10; * *p* < .05; ** *p*. 01 (two-tailed)

^a^Average effects for control variables: age (Coeff = −.1.0524*** *SE* = .006), age^2^ (Coeff. = .107***, *SE* = .007), education (incomplete primary = 0, primary = 1, Coeff = .083, *SE* = .078, incomplete secondary vocational = 2, Coeff = .113 + , *SE* = .077, secondary vocational = 3, Coeff = .188**, *SE* = .008, incomplete secondary academic = 4, Coeff = .221***, *SE* = .008, secondary academic = 5, Coeff = .250***, *SE* = .076, incomplete university = 6, Coeff = .330***, *SE* = .078, university = 7 Coeff = .395***, *SE* = .078), church attendance (Coeff = .046***, *SE* = .005), marital status (married/living together, single, Coeff = −.505***, SE = .026, divorced or separated, Coeff = −.713***, *SE* = .035, widowed, Coeff = −.962***, *SE* = .074), income (Coeff = .080***, *SE* = .004)

^b^Average effects for control variables: GDP (natural logarithm) (Coeff = .397***, *SE* = .417)

Model 2 then retests the social norm to work theory (e.g. Stavrova et al. 2011; Stam et al. 2016) with our new measure for the social norm to work. It confirms the social norm to work theory and shows that the negative effect of being unemployed on the level of well-being is more pronounced in societies with a stronger norm to work. For men, each unit increase in the popular endorsement of the social norm to work increases the well-being disadvantage of being unemployed by .185 points (on a 0–9 scale, *p* = .002). The well-being disadvantage of unemployed men is .683 points^4^ larger in the country with the strongest norm to work (7.93 in Slovenia in 1992) as compared with the weakest (4.24 in Estonia in 1990). For women, however, the corresponding difference between the two countries at the extreme is only .289 points.^5^ Each unit increase in the popular endorsement of the social norm to work increases the well-being disadvantage of female unemployed by only .078 points (*p* = .144). Rather, it is female students who are less well off in societies with a stronger norm to work. The well-being disadvantage of female students (compared to with that of the employed) increases by .175 (*p* = .009) with each unit increase in the popularity of the social norm to work.

Model 3 controls for societies’ income replacement rates. Although this seriously reduces the number of countries under study (to 30 countries, and 41 country-years), it leaves the main results intact.

Model 4, finally, includes societies’ work ethic and its potential interaction effect with employment status. The number of country-years is further reduced (to 38 country-years). Work ethic has been used in previous research as a proxy for the social norm to work (e.g. Stavrova et al. 2011; Stam et al. 2016). Model 4 shows that there are no significant interactions between work ethic and the well-being impact of unemployment, except for a marginally statistically significant positive interaction effect (*b* = .316, *p* = .085) for women. Students appear to have a higher level of well-being (compared to the employed) in societies with a stronger work ethic (*b* = .492, *p* = .027 for men; b = .569, *p* = .016 for women). A stronger work ethic appears to enlargen the well-being disadvantage of female retirees. Controlling for this also does not change the result. Indeed, the coefficient for the interaction with our measure of the social norm to work becomes stronger (*b* = −.305, *p* = .001 for male unemployed and *b* = .321, *p* < .001 for women).

### Results from the ESS Dataset

Table 4 presents the outcomes of the multilevel models based on the ESS, focusing on only European countries. The novelty of these models is that we can make a distinction between the short and long term unemployed.

**Table 4:** Multilevel regression on well-being, ESS data

	Men				Women				
	M1	M2	M3	M4	M1	M2	M3		M4
Constant	.516 (.003)***	.516 (.003)***	.500 (.003)***	.502 (.003)***	.555 (.003)***	.555 (.003)***	.540 (.003)***		.542 (.003)***
*Individual level* ^a^									
Employment status (ref = employed)									
Unemployed short	−.819 (.059)***	−.825 (.064)**	−.779 (.075)**	−.635 (.805)*	−.598 (.057)***	−.605 (.057)***	−.635 (.060)***		−.806 (.870)
Unemployed long	−1.253 (.069)***	−1.160 (.079)**	−.962 (.097)**	−.674 (.978)	−.681 (.060)***	−.680 (.060)***	−.674 (.065)***		−1.485 (.865)^+^
In education	.164 (.054)**	.163 (.062)**	.112 (.072)	−2.410 (.703)**	.194 (.047)***	.195 (.047)***	.161 (.049)**		−2.132 (.635)**
Non-working disabled	−.950 (.061)***	−.965 (.070)**	−.918 (.081)**	1.421 (.769)^+^	−.870 (.057)***	−.867 (.057)***	−.890 (.060)***		−.891 (.706)
Retired	−.112 (.050)*	−.123 (.055)*	−.046 (.063)	3.312 (.652)***	−.111 (.043)*	−.110 (.043)*	−.102 (.047)*		3.940 (.616)***
Homemaker	−.074 (.048)	−.081 (.056)	−.141 (.073)^+^	1.183 (.624)^+^	−.015 (.033)	−.013 (.033)	−.016 (.035)		.471 (.439)
Other	−.186 (.064)***	−.214 (.077)**	−.178 (.091)^+^	−.632 (.882)	−.059 (.057)	−.047 (.058)	−.043 (.062)		−.174 (.744)
*National level* ^b^									
Norm to work	−.109 (.122)	−.111 (.128)	−.017 (.132)	−.069 (.127)	−.149 (.099)	−.095 (.103)	−.033 (.109)		−.086 (.103)
Unemployment rate	−.057 (.012)***	−.056 (.012)**	−.054 (.013)**	−.062 (.016)***	−.037 (.010)***	−.037 (.010)***	−.031 (.011)***		−.048 (.013)***
Replacement rate			−.011 (.004)**	−.007 (.005)			−.007 (.003)*		−.001 (.005)
Work ethic				.599 (.365)					
*Cross-level interactions*									
Norm to work*									
Unemployed short		.021 (.094)	−.079 (.120)	−.152 (.122)		−.191 (.096)*	−.243 (.123)*		−.260 (.127)*
Unemployed long		−.281 (.115)*	−.621 (.154)***	−.473 (.164)**		−.051 (.104)	−.322 (.143)*		−.346 (.149)*
In education		.005 (.087)	.075 (.109)	.000 (.113)		−.058 (.075)	.003 (.096)		−.061 (.099)
Non-working disabled		.049 (.106)	−.102 (.134)	.040 (.135)		−.117 (.103)	−.163 (.132)		−.197 (.136)
Retired		.034 (.076)	−.122 (.096)	.011 (.097)		−.015 (.061)	−.057	(.085)	.081 (.086)
Homemaker		.016 (.080)	.081 (.112)	.111 (.119)		−.063 (.055)	−.068 (.072)		−.102 (.070)
Other		.069 (.110)	.040 (.140)	.065 (.147)		−.147 (.108)	−.185 (.133)		−.238 (.141)^+^
Work ethic*				−.007 (.005)					
Unemployed short				.321 (.230)					.048 (.246)
Unemployed long				−.680 (.282)*					.233 (.243)
In education				.730 (.203)***					.656 (.181)***
Non-working disabled				−.676 (.224)**					.002 (.205)
Retired				−.962 (.187)***					−1.138 (.173)***
Homemaker				−.384 (.183)*					−.142 (.125)
Other				−.233 (.238)					.038 (.214)
Respondents	58,474	58,474	52,657	47,963	66,759	66,759	58,854		53,983
Country-years	91	91	80	74	91	91	80		74
Countries	31	31	27	26	31	31	27		26

Data are nested in country-years nested in countries. In Model 2 and 3 a random slope for employment status is included. Year fixed effects are included, but not presented

^a^Average effects for control variables: age (Coeff = −.972*** *SE* = .051), age^2^ (Coeff. = .099***, *SE* = .006), education (less than lower secondary = 1, lower secondary = 2, Coeff = .110**, *SE* = .043, lower tier upper secondary = 3, Coeff = .151***, *SE* = .040, upper tier upper secondary = 4, Coeff = .263***, *SE* = .043, advanced vocational sub-degree = 5, Coeff = .298***, *SE* = .052, lower tertiary = 6, Coeff = .290***, *SE* = .044, higher tertiary = 7, Coeff = .325***, *SE* = .044), church attendance (Coeff = .079***, *SE* = .006), marital status (married/living together, separated, Coeff = −.828***, SE = .057, divorced, Coeff = −.503***, *SE* = .012, widowed, Coeff = −.742***, *SE* = .063, never married, Coeff = −.430***, SE = .021), income (Coeff = .358***, *SE* = .010)

^b^Average effects for control variables: GDP (natural logarithm) (Coeff = .353, *SE* = .419)

Model 1 again shows the main effects of the individual- and national-level variables. It shows, for both men and women, that the well-being of the unemployed, disabled, retired, and ‘other’ group is lower than that of the employed. As expected, in particular the well-being of the unemployed is lower. Among the unemployed, the long-term unemployed are less well off than the short-term unemployed. Furthermore, the disabled are also at a large well-being disadvantage, even more so than the unemployed among females. For the control variables, again the well-known effects are observed: a higher education level, religiosity, being married/living together, and a higher income level were related to higher levels of well-being. Age is related to a lower level of well-being and has the well-known u-shape. Also in the ESS, a strong norm to work does not influence average levels of well-being. Countries’ wealth has no impact on general well-being either, but when the unemployment rate is higher, levels of well-being decrease.

Model 2 tests the main hypotheses of this study and shows that the negative well-being effect of being unemployed on the level of well-being is more pronounced in societies with a stronger social norm to work. However, there are important differences between the short- and long-term unemployed, showing how important it is to make this distinction. For men, there is no significant cross-level interaction between being short-term unemployed and the social norm to work. However, in line with the idea that the long-term unemployed are more affected by a strong social norm to work, there is a significant interaction between being long-term unemployed and the social norm to work, indicating that the well-being of the long-term unemployed (compared to the employed) is lower in countries with a stronger social norm to work. For men, this well-being disadvantage increased by .281 points (*p* = .015) with each unit increase in the popularity of the norm to work, compared to only .021 (*p* = .827) for short-term unemployed. This suggests that the well-being disadvantage of unemployed men is .778 points^6^ larger in the ESS-country with the strongest norm to work (Italy in 2002 with a norm to work of 7.55) as compared with the weakest (Ukraine in 2004 with a norm to work of 4.78).

For women, the social norm to work appears to enlarge the well-being disadvantage of the short-term unemployed (b = −.191, *p* = .046) but unexpectedly not of the long-term unemployed (b = −.051, *p* = .622). This indicates the social norm to work is still more pressing on men than on women in most societies.

Model 3 controls for replacement rates. This significantly reduces the number of countries under study (to 27 countries, and 80 country years), but does not affect the main results. Interestingly, this time the well-being disadvantage of long-term unemployment is clearly increased by a stronger social norm to work, both for men and for women. With each 1-unit increase in the social norm to work, the well-being disadvantage for long-term unemployed women (compared to the employed) increases by .322 (*p* = .024). The corresponding interaction effect for men is also bolstered by controlling for the replacement rate: their well-being disadvantage now increases with more than a half point (*b* = −.621, *p* < .001) for each unit increase in the social norm to work. This difference amounts to a .32 standard deviation in well-being. The difference between the countries with the weakest and strongest social norm to work implies a lower level of well-being among long-term unemployed men by 1.719 points (compared to the employed) in the strongest country. This difference amounts to almost one standard deviation, reflecting a modest-to-strong effect.

Model 4, finally, controls for the possibility that the interaction effects are explained by countries’ work ethic. This again reduces the sample size (to 26 countries). Just as in the WVS/EVS data, the main results are unchanged. Work ethic itself has also some statistically significant interaction effects with employment status. For instance, work ethic increases the well-being disadvantage of long-term unemployed men (*b* = −.680, *p* = .016), disabled men (*b* = −.676, *p* = .003), the retired (*b* = −.384, *p* = .036 for men and *b* = −1.138, *p* < .001 for women) and increases the well-being advantage of students (*b* = .730, *p* < .001 for men and *b* = .656, *p* < .001 for women).

### Robustness Checks

We conducted several additional robustness checks. First, we tested whether the interaction between the norm to work and employment status is not explained solely by other measures of the norm to work (in interaction with employment status): unemployment rates and replacement rates, respectively. Because employment status has seven categories, this yields (3 × 7 = 21) extra interactions. Importantly, despite taking these variables into account, the effects of the norm to work on the impact of unemployment are unchanged and even occasionally increase in size.

A supplemental robustness check controlled for a potential interaction effect with countries’ work ethic (as in Model 4), but without controlling for replacement rate. This slightly increases the sample size (to 40 countries and 80 country-years in the WVS, and 30 countries and 85 country-years in the ESS). In the WVS, our measure of the norm to work still strengthens the effect of unemployment, whereas countries’ work ethic does not. However, for the ESS the interaction effect lost statistical significance when including the work ethic interaction term. The different number of countries and leaving replacement rates out of the model may explain these differences, but we find it most likely that it is due to the low statistical power (because of the small n on the country level) in combination with running two cross-level interactions. This can be a little overdemanding the data (e.g. Bryan and Jenkins 2016). This indicates that we have to be careful in stating that there is an effect of our measure of the social norm to work and that it is stronger than the effect of the work ethic measure.

### Comparing the WVS/EVS with the ESS

In a further step, in order to compare the results from the WVS/EVS dataset with those of the ESS dataset, we examined only European countries in the WVS/EVS. To compare the outcomes of the WVS/EVS with those of the ESS, we rescaled the well-being question in the EVS/WVS to a 10–point scale and only selected European countries, and we merged the short- and long-term unemployed in the ESS. We included the same control variables as in Model 3.

The effects of the norm to work on the level of well-being of the unemployed are slightly stronger in the WVS/EVS if we include all countries (*b* = −.291, *p* = .005 for men; *b* = −.248, *p* = .040 for women) than when we include only European countries (*b* = −.265, *p* = .022 for men; *b* = −.221, *p* = .080 for women). One possible explanation is that the large welfare states in Europe might protect their unemployed from being affected by a strong social norm to work. The figures for men are remarkably similar to those in the ESS (*b* = −.275, *p* = .008). For women, they are slightly higher in the ESS (*b* = −.283, *p* = .006). This may be because we did not control for individual’s norm to work in the ESS, and women may be more affected by this than men. Alternatively, it may simply be explained by random noise, as the figures in the WVS/EVS are of approximately the same magnitude as in the ESS.

## Conclusion and Discussion

According to the social norm theory of unemployment, the well-being of the non-employed is lower in countries with a strong social norm to work because of the greater stigma attached to unemployment (Clark 2003; Oesch and Lipps 2013; Stam et al. 2016; Stavrova et al. 2011).

Our first step was retesting this theory with an alternative measure of the social norm to work that directly assesses whether people expect others to behave in conformity with their personal norm, and this measure is available for a large number of countries. The results support the theory and underscore the importance of a strong social norm to work in accounting for differences in the relative well-being of unemployed people across countries, in particular for men. The magnitude of this effect remained strong, even after controlling for alternative explanations and mechanisms, such as individual income, unemployment rates, personal subscription to the norm to work, and individual and national work ethic. Hence, the norms that people impose on the non-employed lower their levels of well-being, potentially accompanied by non-pecuniary social sanctions, such as social disapproval and rejection. This contradicts the wider claim that the external enforcement of social norms has faded in modern societies (Elias 1982; Heckhausen 1999). However, contrary to what was expected, the results show there is little evidence that the well-being of the economically inactive is lower in countries with a stronger social norm to work. Hence, it seems that these groups may be less exposed to a social norm to work than previously argued (Stam et al. 2016).

Furthermore, we built on previous research by showing that there is a distinction between the short- and long-term unemployed. The level of well-being of the long-term unemployed is typically lower than that of the short-term unemployed; and this is particularly so in societies with a strong social norm to work. The gap between the employed and the long-term unemployed is approximately .78 (men) and .14 (for women) unit-points larger in the countries with the strongest social norm to work in comparison with the weakest. For men, this is a large gap, given that the majority of respondents had well-being scores between 6 and 9 on this well-being scale. Especially the long-term unemployed may be disqualified in societies with a strong social norm to work and may feel uncomfortable about not working, because they can more easily be seen as being lazy and not willing to work, while the short-term, possibly temporarily, unemployed can more readily overcome their unemployment. Our findings fit with previous qualitative findings that also indicated that the long-term unemployed are aware of these negative, individual attributions of their status (Kampen et al. 2013). This indicates that previous studies that grouped the short- and long-term unemployed together have probably underestimated the effects of a social norm to work (e.g. Gallie and Russell 1998; Stam et al. 2016; Stavrova et al. 2011).

The fact that we find larger effects of the norm to work on (long-term unemployed) men than on women indicates that this norm is still more pressing on men than on women in most societies (Gallie and Russell 1998; Stam et al. 2016; Van der Meer 2014). However, studies have also indicated that women in some countries face more pressure to work and less access to equally accepted alternative roles such as the homemaker than in other countries (Gallie and Russell 1998; Whelan and McGinnity 2000). This larger acceptability of the homemaker role (and of other economically inactive roles) for women, could also explain why we do not find a social norm effect on long-term unemployed women: long-term unemployed may be more prone to self-categorize as other ‘economically inactive’. Increasing women’s emancipation only in the narrow form of corporate feminism could lead to a strengthening of the social norm to work for women in the future (Strandh et al. 2013). Although we tried to measure a social norm to work more precisely than previous research has done, it needs to be further specified in future research. In this study, a social norm to work is regarded as a national characteristic. Although this makes sense because people are often confronted with national sentiments, for instance through the media, norms might apply stronger for certain groups or in specific networks and are often enforced in individual settings (Clark 2003; Hedström 2005). For example, a social norm to work may be stronger for younger than for older people because one may think that it is difficult for older people to obtain a job and its effect on well-being may be stronger for people whose friends subscribe to this norm than for those whose friends accept that one is unemployed.

Furthermore, we have to note that we should be careful by overemphasizing the pressure on long-term unemployed as these results are a bit unstable in the European context—i.e. they remain strong but become not significant—once we add additional cross-level interactions between work ethic and work status in the ESS data. However, we should keep in mind that including multiple cross-level interaction on a dataset with relatively few countries demands a lot from the data, and soon can result in instable effects, particularly once focusing solely on the significance levels (e.g. Bryan and Jenkins 2016). Nonetheless, further research is needed to establish the strength of this relationship.

Future research is also needed to more specifically examine the effects on the short and long-term unemployed. We used a rather crude binary distinction between short- and long-term unemployment. Future research could implement a more refined measure. For instance, the effects of the social norm to work may have a u-shaped effect, becoming gradually stronger when people have been unemployed longer, but flattening out for people who have been unemployed a very long time as they may get used to it. Furthermore, individual characteristics of the unemployed may be taken into account, for instance their wealth. This is important as people can use their wealth to temporarily compensate their losses in income.

In contrast to previous research, we do not find that the well-being of other economically inactive groups, such as homemakers, is affected by the social norm to work. However, this group is very heterogeneous, and some individuals counted as inactive may be ‘hidden unemployed’ for whom the social norm to work still largely applies. Alternatively, many non-working disabled respondents may have incorporated the dominant idea that they can now work and now self-identify as unemployed. Future research is needed to better distinguish these groups from people who are economically inactive by choice.

Despite these limitations, we showed that the social norm to work theory holds across a wider span of countries and years, and we used a new and novel measure to capture social expectations towards others. Furthermore, we extended the social norm to work theory by showing that a distinction between the short- and long-term unemployed has to be made as the social norm to work is more pressing on the short-term unemployed women and long-term unemployed men.

## Appendix 1

See Table 5.

**Table 5:** Robustness multilevel regression on well-being, WVS/EVS data

	Men		Women	
	M4	M5	M4	M5
Constant	.477 (.005)***	.477 (.005)***	.476 (.005)***	.476 (.005)***
*Individual level*				
Employment status (ref = full-time)				
Unemployed	.473 (.619)	.837 (.620)	−.789 (.199)***	.032 (.202)
Retired	.517 (.532)	.141 (.557)	.011 (.182)	−.338 (.182)^+^
Housewife	−4.757 (2.435)^+^	−4.339 (2.507)^+^	−.131 (.161)	−.071 (.162)
Students	1.795 (.900)*	2.834 (.847)**	−.288 (.238)	.229 (.238)
Part-time	−.301 (.900)	.548 (.906)	−.045 (.169)	.068 (.184)
Self-employed	1.295 (.544)*	1.048 (.556)^+^	.143 (.232)	.107 (.232)
Other	.519 (.996)	.442 (1.074)	−1.254 (.267)***	−1.033 (.294)***
Norm to work	.033 (.006)***	.033 (.006)***	.026 (.006)***	.026 (.006)***
*Individual level interactions*				
Norm to work*				
Unemployed	.007 (.017)	.009 (.017)	−.008 (.018)	−.007 (.018)
Retired	−.013 (.017)	−.012 (.017)	.006 (.017)	.006 (.017)
Housewife	.056 (.062)	.054 (.062)	−.005 (.012)	−.005 (.012)
Students	−.011 (.026)	−.008 (.026)	.041 (.024)^+^	.043 (.024)^+^
Part-time	.011 (.030)	.011 (.030)	.003 (.015)	.003 (.015)
Self-employed	−.020 (.016)	−.018 (.016)	.013 (.022)	.013 (.022)
Other	.072 (.028)*	.072 (.028)*	.058 (.025)*	.059 (.025)*
*National level variables*				
Norm to work	−.493 (.301)	−.464 (.317)	−.326 (.350)	−.269 (.341)
Unemployment rate	.035 (.024)	.041 (.024)	.030 (.029)	.036 (.027)
Replacement rate	−.008 (.006)	−.008 (.006)	−.003 (.007)	.001 (.007)
*Cross-level interactions*				
Norm to work*				
Unemployed	−.249 (.091)**	−.271 (.093)**	−.197 (.107)^+^	−.290 (.105)**
Retired	−.085 (.080)	−.057 (.082)	.078 (.095)	.107 (.093)
Housewife	.667 (.377)^+^	.670 (.378)^+^	.093 (.093)	.072 (.089)
Students	−.298 (.128)*	−.374 (.125)**	−.150 (.128)	−.207 (.126)^+^
Part-time	−.044 (.137)	−.094 (.137)	−.028 (.101)	−.038 (.099)
Self-employed	−.156 (.080)^+^	−.136 (.082)^+^	.175 (.121)	.185 (.118)
Other	−.249 (.150)^+^	−.250 (.155)	.291 (.152)^+^	.295 (.155)^+^
Unemployment rate*				
Unemployed	.024 (.015)		.038 (.020)^+^	
Retired	−.008 (.013)		−.023 (.017)	
Housewife	.026 (.074)		.027 (.018)	
Students	.030 (.021)		.018 (.023)	
Part-time	.040 (.021)^+^		.003 (.018)	
Self-employed	−.017 (.014)		−.024 (.023)	
Other	−.019 (.025)		.043 (.028)	
Replacement rate*				
Unemployed		−.001 (.004)		−.014 (.004)**
Retired		.004 (.003)		.005 (.004)
Housewife		−.007 (.015)		.004 (.004)
Students		−.009 (.004)*		−.010 (.004)*
Part-time		−.007 (.005)		−.002 (.004)
Self-employed		.001 (.003)		−.004 (.005)
Other		−.001 (.006)		.002 (.006)
Respondents	19,464	19,464	23,259	23,259
Country-years	41	41	41	41
Countries	30	30	30	30

Data are nested in country-years nested in countries. All models include a random slope for employment status

Year fixed effects are included, but not presented. Unstandardized results, standard errors in parentheses

These analyses also controlled for age, age squared, educational level, church attendance marital status, income and GDP. (a) Work ethic was measured using the same measurement as in Stam et al. (2016) and Stavrova et al. (2011), using people’s endorsement of the following five statements: ‘Work is a duty towards society’ ‘People who don’t work turn lazy’, ‘It is humiliating to receive money without having to work for it’, ‘Work should always come first, even if it means less spare time’, and ‘To fully develop your talents, you need to have a job’

+ *p* < .10; * *p* < .05; ** *p* .01 (two-tailed)

## Appendix 2

See Table 6.

**Table 6:** Robustness multilevel regression on well-being, ESS data

	Men		Women	
	M4	M5	M4	M5
Constant	.500 (.003)***	.500 (.003)***	.540 (.003)***	.540 (.003)***
*Individual level* ^a^				
Employment status (ref = employed)				
Unemployed short	−.859 (.143)***	−.819 (.163)***	−.646 (.154)***	−.842 (.169)***
Unemployed long	−.880 (.170)***	−1.153 (.205)***	−.760 (.153)***	−.673 (.173)***
In education	−.213 (.125)^+^	.649 (.147)***	−.054 (.107)	.627 (.128)***
Non-working disabled	−.509 (.155)**	−.948 (.180)***	−1.055 (.153)***	−1.053 (.171)***
Retired	.079 (.110)	−.378 (.128)***	.267 (.097)**	−.529 (.107)***
Homemaker	.021 (.108)	−.411 (.146)**	.071 (.075)	−.137 (.087)
Other	.041 (.154)	−.333 (.193)^+^	−.001 (.136)	.040 (.173)
*National level*				
Norm to work	−.014 (.123)	−.024 (.122)	−.030 (.104)	−.024 (.122)
Unemployment rate	−.048 (.014)	−.053 (.012)***	−.024 (.012)*	−.053 (.012)***
Replacement rate	−.011 (.004)**	−.012 (.004)**	−.007 (.003)*	.001 (.001)
Work ethic				
*Cross-level interactions*				
Norm to work*				
Unemployed short	−.077 (.115)	−.075 (.114)	−.241 (.121)*	−.237 (.119)*
Unemployed long	−.624 (.150)***	−.607 (.149)***	−.331 (.140)*	−.321 (.139)*
In education	.104 (.104)	.071 (.103)	.019 (.093)	−.005 (.092)
Non-working disabled	−.123 (.129)	−.079 (.128)	−.145 (.129)	−.149 (.128)
Retired	−.129 (.090)	−.089 (.090)	−.066 (.081)	−.015 (.080)
Homemaker	.081 (.106)	.122 (.107)	.072 (.068)	−.058 (.066)
Other	.038 (.135)	.061 (.136)	−.188 (.130)	−.216 (.133)
Unemployment rate*				
Unemployed short	.009 (.016)		.001 (.018)	
Unemployed long	−.011 (.018)		.009 (.017)	
In education	.043 (.014)**		.030 (.013)*	
Non-working disabled	−.059 (.019)**		.026 (.022)	
Retired	−.017 (.012)		−.049 (.011)***	
Homemaker	−.023 (.012)^+^		−.012 (.009)	
Other	−.032 (.019)^+^		−.005 (.019)	
Replacement rate*				
Unemployed short		.001 (.004)		.005 (.004)
Unemployed long		.005 (.004)		−.001 (.004)
In education		−.013 (.003)***		−.011 (.003)***
Non-working disabled		.001 (.004)		.004 (.004)
Retired		.008 (.003)**		.011 (.002)***
Homemaker		.006 (.003)*		.003 (.002)
Other		.004 (.004)		−.002 (.004)
Respondents	52,657	52,657	58,854	58,854
Country-years	80	80	80	80
Countries	27	27	27	27

Data are nested in country-years nested in countries. All models include a random slope for employment status

Year fixed effects are included, but not presented. Unstandardized results, standard errors in parentheses

These analyses also controlled for age, age squared, educational level, church attendance marital status, income and GDP

+ *p* < .10; * *p* < .05, ** *p* .01 (two-tailed)

^a^Work ethic was measured using the same measurement as in Stam et al. (2016) and Stavrova et al. (2011), using people’s endorsement of the following five statements: ‘Work is a duty towards society’, ‘People who don’t work turn lazy’, ‘It is humiliating to receive money without having to work for it’, ‘Work should always come first, even if it means less spare time’, and ‘To fully develop your talents, you need to have a job’

## Footnotes

1 WVS/EVS: Austria (1990, 1999, 2008), Belgium (1990, 1999, 2009), Canada (1990), Czech Republic (1991, 1998, 1999, 2008), Denmark (1990, 1999, 2008), Estonia (1990, 1996, 1999, 2008), Finland (1990, 1996, 2000, 2005, 2009). France (1990, 1999, 2006, 2008), Germany (1990, 1997, 1999, 2006, 2008), Greece (1999, 2008), Hungary (1991, 1998, 1999, 2008), Iceland (1990, 1999, 2009), Ireland (1990, 1999, 2008), Italy (1990, 1999, 2005, 2009), Japan (1990), Luxembourg (1999, 2008), Mexico (1990), Netherlands (1990, 1999, 2006, 2008), Norway (1990, 1996, 2007, 2008), Poland (1990, 1999, 2005, 2008), Portugal (1990, 1999, 2008), Slovakia (1991, 1998, 1999, 2008), Slovenia (1992, 1995, 1999, 2005, 2008), Spain (1990, 1995, 1999, 2000, 2007, 2008), Sweden (1990, 1996, 1999, 2006, 2009), Switzerland (2008), Turkey (2009), Great Britain (1990, 1998, 1999, 2005, 2009), United States (1990), Croatia (1999, 2008), Bulgaria (1991, 1997, 1999, 2005, 2008), Romania (1993, 1998, 1999, 2005, 2008), Latvia (1996, 1999, 2008), Lithuania (1997, 1999, 2008), Cyprus (2008), Malta (1999, 2008), Albania (2008), Russia (1995, 1999, 2006, 2008), Ukraine (1999, 2006, 2008), Argentina (1991), Brazil (1991), Bosnia (2008), Belarus (2000, 2008), Georgia (2008), Moldova (2008), Montenegro (2008), Serbia (2008), Macedonia (2008). ESS: Austria (2002, 2004, 2006), Belgium (2002, 2004, 2006, 2008), Czech Republic (2002, 2004, 2008), Denmark (2002, 2004, 2006, 2008), Estonia (2004, 2006, 2008), Finland (2002, 2004, 2006, 2008), France (2002, 2004, 2006, 2008), Germany (2002, 2004, 2006, 2008), Greece (2002, 2004, 2008), Hungary (2002, 2004, 2006, 2008), Iceland (2004), Ireland (2002, 2004, 2006, 2008), Italy (2002), Luxembourg (2002, 2004), Netherlands (2002, 2004, 2006, 2008), Norway (2002, 2004, 2006, 2008), Poland (2002, 2004, 2006, 2008), Portugal (2002, 2004, 2006, 2008), Slovakia (2004, 2006, 2008), Slovenia (2002, 2004, 2006, 2008), Spain (2002, 2004, 2006, 2008), Sweden (2002, 2004, 2006, 2008), Switzerland (2008), Great Britain (2002, 2004, 2006, 2008), Croatia (2008), Bulgaria (2006, 2008), Romania (2008), Latvia (2008), Cyprus (2008), Russia (2006, 2008), Ukraine (2004, 2006, 2008).

2 It is not entirely clear to us why the current measure and the work-ethic correlate so weakly. Part of the explanation may lie in the fact that the popular measure of work ethic does not only ask about questions that are related to the unemployed. This conjecture is supported by the fact that the correlation between the current measure and items on the work ethic that do not directly ask about the unemployed is weakest. Alternatively, it could be indeed the case that individuals that hold a strong work ethic for themselves do not necessarily impose this norm on others.

3 This was not possible in the ESS, as these models did not converge. For the sake of thoroughness, we also ran models with random effects, because adding country-fixed effects takes away all (including theoretically interesting) variance between countries (Beck 2001) and the largest variation in the social norm to work is cross-national and not cross-temporal. This did substantially alter the results. Estimates can be requested from the author.

4 The coefficient of −.185* the maximal range in countries’ social norm to work.

5 The coefficient of −.078* the maximal range in countries’ social norm to work.

6 The coefficient of −.281* the maximal range in countries’ social norm to work.
